# Higher Urinary Sodium, a Proxy for Intake, Is Associated with Increased Calcium Excretion and Lower Hip Bone Density in Healthy Young Women with Lower Calcium Intakes

**DOI:** 10.3390/nu3110951

**Published:** 2011-11-10

**Authors:** Jennifer L. Bedford, Susan I. Barr

**Affiliations:** 1 Diabetes Education Centre, Ross Memorial Hospital, 10 Angeline St N, Lindsay, Ontario K9V 4M8, Canada; Email: jennifer.lynn.bedford@gmail.com; 2 University of British Columbia, 2205 East Mall, Vancouver, BC V6T 1Z4, Canada

**Keywords:** urinary sodium, urinary calcium, bone mineral density, premenopausal women

## Abstract

We assessed 24-h urinary sodium (Na) and its relationship with urinary calcium (Ca) and areal bone mineral density (aBMD) at the whole body, lumbar spine and total hip in a cross-sectional study. 102 healthy non-obese women completed timed 24-h urine collections which were analyzed for Na and Ca. Dietary intakes were estimated using a validated food frequency questionnaire. Participants were grouped as those with lower *vs*. higher calcium intake by median split (506 mg/1000 kcal). Dietary Na intake correlated with 24-h urinary loss. Urinary Na correlated positively with urinary Ca for all participants (*r* = 0.29, *p* < 0.01) and among those with lower (*r* = 0.37, *p* < 0.01) but not higher calcium intakes (*r* = 0.19, *p* = 0.19). Urinary Na was inversely associated with hip aBMD for all participants (*r* = −0.21, *p* = 0.04) and among women with lower (*r* = −0.36, *p* < 0.01) but not higher (*r* = −0.05, *p* = 0.71) calcium intakes. Urinary Na also entered a regression equation for hip aBMD in women with lower Ca intakes, contributing 5.9% to explained variance. In conclusion, 24-h urinary Na (a proxy for intake) is associated with higher urinary Ca loss in young women and may affect aBMD, particularly in those with lower calcium intakes.

## 1. Introduction

Whether sodium intake has an impact on bone health has been studied since Goulding observed that sodium intake affected bone mass in animals [1,2]. Since then, most studies have observed significant correlations between 24-h measures of urinary calcium and sodium [3,4,5]. This is because renal calcium reabsorption is influenced by the concurrent degree of sodium reabsorption [6]. Most evidence supports an increase in urinary calcium of about 1 mmol per 100 mmol of sodium excretion, which equates to an increase of 40 mg of calcium excreted in the urine for every 2300 mg of dietary sodium, although variability does exist [3,4,5]. 

Over time, sodium-induced calciuria may have the potential to negatively impact bone mineral density (BMD) [3,4,5,7]. It has been suggested by Heaney [3] that the impact of sodium on bone may depend on adequacy of calcium intake: at recommended calcium intakes, typical North American sodium intakes would have little effect on calcium balance; however, at low calcium intakes the adaptive increase in intestinal calcium absorption may be insufficient to offset the additional urinary calcium losses. Data are not consistent with regard to whether the association differs at low *versus* high calcium intakes. Urinary sodium and calcium were associated at high but not low calcium intakes among healthy elderly persons [8]. Conversely, in studies of postmenopausal women, urinary sodium and calcium were associated at low but not high calcium intakes [9,10]. Few data are available regarding whether calcium intake modulates the relationship between urinary calcium and sodium in young women. 

We recently conducted a 2-year study of dietary attitudes, ovulatory function, and bone density in healthy young women [11]. At the 2-year follow-up, 24-h urine collections were analyzed for sodium and calcium. Herein, we report the cross-sectional relationship between 24-h urinary sodium and urinary calcium excretion, and examine whether the association differs between those with higher *versus* lower calcium intakes. Associations with BMD and 2-year BMD change were also assessed. 

## 2. Methods

### 2.1. Participant Characteristics

As described in detail elsewhere [11], interested women were recruited between August and December 2006 and were screened for eligibility by telephone interview. Eligibility criteria were: 19-35 years old, regular menses (self-reported menses every 21-35 days in the previous ≥6 months), non-obese (self-reported body mass index (BMI) 18-30 kg/m^2^), no pregnancy/breastfeeding currently or within 12 months, and absence of medical conditions (current or previous diagnosis of eating disorder, polycystic ovarian syndrome, Cushing’s syndrome, inflammatory conditions, hypertension, hyperthyroidism or hirsutism) or use of medications (oral contraceptives, progesterone or glucocorticoids currently or within the past 6 months) that could affect study variables. Of 148 women assessed, 142 were eligible, 137 completed baseline data collection and 123 completed the final follow-up (*n* = 4 moved, *n* = 5 no longer wanted to participate or did not respond, *n* = 5 became ineligible due to pregnancy, androgen excess, thyroid cancer). Among this group, 110 had complete data from a food frequency questionnaire (FFQ), 24-h urine collection and dual energy X-ray absorptiometry (DXA) scan at the final follow-up, which was the only time point at which urinary sodium and calcium were assessed. The study protocol was approved by the university’s Clinical Research Ethics Board (protocol C05-0257) in June 2005 and renewed annually through June 2009. Written informed consent was obtained from all participants. 

### 2.2. Urine Collection and Analysis

Participants met with an investigator to receive materials and oral and written instructions for home completion of a 24-h urine collection. Participants were instructed to complete the urine collection within several weeks of the meeting, on a “normal day” free of any unusual physical or mental stresses, after reviewing written instructions. On the day of collection, participants discarded their first urine void, recorded the time this occurred and then collected all subsequent voids for 24 h including a void at the recorded time the following morning. Urine collections were delivered by courier to the laboratory, where volume was measured and aliquots were frozen and stored prior to analysis of urinary sodium, calcium and creatinine using a calibrated Dade Behring Dimension RXL (Siemans AG, Munich, Germany) automated clinical chemistry analyzer [12]. 

### 2.3. Physical Measurements

Measured height and weight were used to calculate BMI. DXA scans of the lumbar spine (L1-4), both total hips and whole body were completed at baseline and final follow-up. Total body bone-free lean body mass (LBM, kg), fat mass (kg), and areal BMD (aBMD, g/cm^2^) were measured on a Lunar Prodigy machine with enCORE^®^ software (GE Healthcare, Madison, WI). Daily quality assurance tests were conducted using a spine phantom scan and densitometric calibration. The in-house coefficient of variation (CV) with repositioning for aBMD at L1-4 averaged 0.94% (0.82-1.10%) and the CV for total proximal femur averaged 0.70% (0.65-0.76%). The 2-year bone measurements were conducted 1.95 ± 0.14 year after baseline. Measurements before or after the 2-year time point were corrected to 2-year percent change. 

### 2.4. Food Frequency Questionnaire

To determine usual dietary intake, the Diet History Questionnaire [13] was completed. Scannable questionnaires were analyzed with a Canadian version of the nutrient database [14]. Participants were classified as those with lower (506 mg/1000 kcal or less) and higher (>506 mg/1000 kcal) calcium intake (from the combination of food and supplements) by median split.

### 2.5. Other Questionnaires

As physical activity is positively associated with BMD, participants’ usual activity at work, in sport, and during leisure was determined using the Baecke Questionnaire of Habitual Physical Activity [15]. Sport and total activity scores are reported. The duration of any reproductive hormone use prior to study entry was documented at baseline, and duration of reproductive hormone use during the study was documented at the final follow-up.

### 2.6. Statistical Analyses

Data were coded, verified, and entered into SPSS software (v.17, SPSS Inc., 2008), and crosschecked for accuracy. Physiologic variables were examined for outliers (mean ± > 4SD) and two individuals with extreme values for urine volume were removed. Completeness of 24-h urine collections was assessed based on expected creatinine excretion (mg/kg body weight) [16]; six individuals with values below or above the expected range were excluded for a final sample size of 102 women.

Descriptive statistics were used to characterize the sample. Differences by calcium intake per 1000 kcal (median split) were examined using Chi-square, *t*-tests and General Linear Model with appropriate covariates. Pearson’s correlation analyses were conducted to examine relationships among dietary and urinary calcium and sodium, and between urinary sodium and cross-sectional and prospective aBMD measures. To examine whether urinary sodium contributed to variance in aBMD independently of other BMD correlates, we conducted linear regression analysis. Variables available for entry into the models were selected based on significant univariate correlations with aBMD measures, and absence of significant collinearity. Analyses were repeated among women with higher and lower calcium intake (median split). All cases were examined pairwise. Results were considered significant at *p* < 0.05. 

## 3. Results

### 3.1. Participants

Participants’ age, physical activity level, reproductive hormone use, urine analyses, physical measurements and dietary intake are described in Table 1. Most participants were single (94%), students (66%) and had completed some post-secondary education (95%). Thirty-four women (33%) had ever used oral contraceptives, for a mean duration of just over two years. Those who used reproductive hormones did not differ in outcome variables (urinary sodium or calcium, or height-and-LBM-adjusted aBMD measures) from non-users (data not shown), and all were included.

Table 1 also describes study variables by the median split of calcium intake. Women with lower calcium intake were slightly shorter, had slightly lower LBM, and were less likely to have ever used reproductive hormones. They also had non-significantly lower urinary creatinine excretion (*p* = 0.066), although when LBM was included as a covariate, the estimated marginal means were almost identical (10.08 ± 1.5 *versus* 10.10 ± 1.5, *p* = 0.937). No differences were observed in age, physical activity, BMI, urinary sodium and calcium excretion, aBMD, or dietary sodium intake.

### 3.2. Urinary and Dietary Sodium and Calcium

Significant correlations were observed between urinary and dietary sodium (*r* = 0.21, *p* = 0.032), but the relationship between urinary and dietary calcium was not significant (*r* = 0.16, *p* = 0.099). Urinary calcium and sodium were significantly correlated (*r* = 0.29, *p* = 0.003). The relationship between urinary calcium and sodium was also apparent among women with lower calcium intake (*r* = 0.37, *p* = 0.008) but not those with higher calcium intake (*r* = 0.19, *p* = 0.186). For the group as a whole, the regression equation generated from the analysis was: Urinary calcium (mg) = 80.5 + (0.019 × urinary sodium (mg)), or alternately: Urinary calcium (mmol) = 2.0 + (0.011 × urinary sodium (mmol)). This indicates that for every 100 mmol (2300 mg) increase in sodium excretion, urinary calcium would be predicted to increase by 1.1 mmol (44 mg). 

**Table 1:** Age, physical activity, reproductive hormone use, 24-h urine analyses, physical measurements and dietary intake among all participants and differences by calcium intake (median split).

	All participants ( *n* = 102)	Calcium intake below median ^1^ ( *n* = 51)	Calcium intake above median ^2^ ( *n* = 51)	*p*
Age (years)	24.0 ± 3.4	23.5 ± 3.0	24.5 ± 3.7	0.119
**Physical activity ^3^**				
Sport activity	2.4 ± 0.8	2.3 ± 0.8	2.4 ± 0.8	0.355
Total activity	7.7 ± 1.5	7.5 ± 1.5	7.8 ± 1.5	0.328
**Reproductive hormone use**				
Ever used (%)	33%	24%	43%	0.036
Duration (months) in users	27.8 ± 28.1	13.8 ± 5.3	35.1 ± 32.4	0.008
**24-h urine analyses**				
Volume (L)	1.66 ± 0.63	1.58 ± 0.60	1.75 ± 0.66	0.179
Calcium (mg)	135.1 ± 68.5	129.3 ± 66.1	141.0 ± 71.0	0.389
Sodium (mg)	2942 ± 1062	2933 ± 1177	2950 ± 946	0.937
Creatinine (mmol)	10.1 ± 2.1	9.7 ± 1.9	10.5 ± 2.2	0.066
**Physical measurements**				
Height (cm)	162.4 ± 6.9	160.8 ± 6.0	163.9 ± 7.5	0.021
Weight (kg)	57.7 ± 8.9	56.8 ± 8.9	58.7 ± 8.9	0.280
BMI (kg/m^2^)	21.8 ± 2.6	21.8 ± 2.9	21.7 ± 2.1	0.819
Lean body mass (kg)	37.6 ± 5.1	36.3 ± 4.4	38.8 ± 5.5	0.014
Fat mass (kg)	17.1 ± 5.4	17.2 ± 5.8	17.0 ± 5.2	0.880
TB aBMD (g/cm^2^)	1.142 ± 0.076	1.133 ± 0.070	1.150 ± 0.082	0.272
L1-4 aBMD (g/cm^2^)	1.190 ± 0.119	1.180 ± 0.097	1.200 ± 0.137	0.384
Hip aBMD (g/cm^2^)	1.020 ± 0.119	1.004 ± 0.109	1.036 ± 0.127	0.170
**Dietary intake ^4^**				
Energy (kcal)	1610 ± 552	1600 ± 587	1620 ± 521	0.858
Protein (g)	65.6 ± 27.9	62.9 ± 28.6	68.2 ± 27.1	0.339
Calcium (mg)	828 ± 390	609 ± 294	1047 ± 351	----
Sodium (mg)	2648 ± 1089	2618 ± 1208	2678 ± 966	0.784

Data are presented as mean ± standard deviation. Differences by calcium intake were examined by independent sample *t*-tests. aBMD, areal bone mineral density; BMI, body mass index; TB, total body; L1-4, lumbar spine vertebrae 1 to 4. ^1^ Calcium intake less than the median (≤506 mg calcium per 1000 kcal). ^2^ Calcium intake greater than the median (>506 mg calcium per 1000 kcal). ^3^ Baecke Habitual Physical Activity Questionnaire [15], possible scores for sport 1-5 and total 3-15. ^4^ Dietary intake assessed by food frequency questionnaire.

### 3.3. Relationships Between Urinary Sodium and aBMD Measures

Table 2 shows cross-sectional associations between urinary sodium and aBMD. Urinary sodium was inversely associated with aBMD at the total hip for the group as a whole. When analyses were conducted separately among those with lower and higher calcium intakes, urinary sodium correlated negatively with aBMD at the total hip among those with lower calcium intakes, and the negative association with whole body aBMD approached significance. No significant associations were seen in those with higher calcium intakes. Urinary sodium was not associated with 2-year aBMD change at any site for all participants or those with higher or lower calcium intakes (data not shown).

**Table 2:** Correlations among urinary sodium and areal bone mineral density in all participants and women with calcium intakes below and above the median.

Site of aBMD	*R*	
	Urinary sodium	*p*
**Total body**		
All participants	−0.14	0.185
Calcium below median ^1^	−0.26	0.069
Calcium above median ^2^	−0.02	0.869
**Lumbar spine**		
All participants	−0.13	0.185
Calcium below median ^1^	−0.19	0.192
Calcium above median ^2^	−0.10	0.495
**Total hip**		
All participants	−0.21	0.039
Calcium below median ^1^	−0.36	0.009
Calcium above median ^2^	−0.05	0.712

Data are presented as Pearson correlation coefficients (R). aBMD, areal bone mineral density (g/cm^2^). ^1^ Calcium intake less than the median (≤506 mg calcium per 1000 kcal). ^2^ Calcium intake greater than the median (>506 mg calcium per 1000 kcal).

In univariate analyses, aBMD measures were associated with height (*r* = 0.33-0.45, *p* < 0.001), weight (*r* = 0.37-0.52, *p* < 0.001), LBM (*r* = 0.52-0.62, *p* < 0.001), sport activity (*r* = 0.31-0.35, *p* < 0.001) and duration of reproductive hormone use (*r* = 0.13-0.25, *p* = 0.009-0.19). Associations with age and dietary calcium intake were not significant. Because associations with LBM were stronger than those with height or weight, and because these three variables were highly intercorrelated, LBM was chosen for availability for the regression models, along with sport activity, duration of reproductive hormone use, and urinary sodium. For the entire group of women, LBM entered regression models for all three aBMD sites (total body, L1-4, and total hip), explaining 39%, 27%, and 32% of the variance, respectively. The hip site was the only aBMD site for which additional variables entered the regression: sport activity score explained an additional 3.3% of the variance, and urinary sodium was narrowly excluded from the model (2.4% of explained variance, *p* = 0.055). Models were also examined for women with lower and higher calcium intakes: urinary sodium entered the equation for total hip aBMD in women with lower calcium intakes (Table 3), but not for those with higher calcium intakes (data not shown). 

**Table 3:** Regression model for hip aBMD in women with calcium intake below the median (*n* = 51).

	*R*^2^ change	*B* ± *S.E.*	*β*	*t*	*p*
(Constant)		0.698 ± 0.117		5.95	<0.001
Lean body mass (kg)	0.241	0.008 ± 0.003	0.305	2.44	0.018
Sport activity score ^1^	0.099	0.043 ± 0.016	0.334	2.73	0.009
Urine sodium (mg)	0.059	−0.001 ± 0.000	−0.251	−2.16	0.036

For the final model, *F* = 10.42, *p* < 0.001. Variable excluded from the model: duration of reproductive hormone use. aBMD, areal bone mineral density. ^1^ Baecke Habitual Physical Activity Questionnaire sport subscale [15].

## 4. Discussion

Numerous studies indicate that higher dietary sodium intake (assessed by 24-h urinary sodium) may increase the excretion of calcium [3,4,5]. Over time, higher urinary calcium losses could negatively impact bone density [7] although few studies have directly examined associations between sodium and bone, and even fewer of these have been conducted in premenopausal women. As it has been suggested that the attainment of peak bone mass may be more relevant to future osteoporosis risk than bone loss in later life [17,18,19], the relationship between sodium and calcium may be particularly relevant in this group. Thus, we assessed potential relationships between 24-h urinary sodium, urinary calcium, aBMD and 2-year aBMD change among 102 non-obese, healthy young women. 

We found significant correlations between urinary sodium and urinary calcium. The magnitude of the association was similar to that previously reported [3,4,5]: for every 100 mmol (2300 mg) increase in sodium excretion, calcium excretion increased by 1.1 mmol (44 mg). In our participants, the relationship was stronger among those with lower calcium intakes and became nonsignificant in women with higher calcium intakes. Our findings are thus consistent with those of Nordin and Polley [9] and Carbone *et al.* [10], who also observed that sodium and calcium excretion were associated at lower but not higher calcium intakes. Carbone *et al.* suggest that sodium-coupled calcium transport in the kidney may predominate at low calcium intakes, but that at higher intakes, sodium-independent mechanisms may become more important, thus decreasing the link between urinary sodium and calcium excretion [10]. However, the mechanism that could underlie this explanation remains to be clarified. In contrast, Dawson-Hughes *et al.* reported that calcium and sodium excretion were correlated at intermediate and high intakes, but not at low intakes [8]. That observation was most consistent among men; in women, the association was significant across all four quartiles of calcium intake, and disappeared only at calcium intakes below 300 mg/day [8]. Virtually all of the participants in the present study had intakes above that level. 

The potential implications of sodium-induced calciuria for bone are likely to be more serious in those with low calcium intakes, who may be unable to increase calcium absorption to fully compensate for increased urinary losses. For example, Heaney [3] noted that to offset the average urinary calcium loss of 1 mmol (40 mg) associated with an increased sodium intake of 100 mmol (2300 mg), gross calcium absorption efficiency would need to increase to 34% (from 25%) in those with intakes of 600 mg/day, and to about 50% (from 37%) in those with intakes of 300 mg/day-and that this may not be possible. However, at intakes of 1200 mg/day, absorption efficiency would only need to increase from to 23% (from 20%) [3]. Empirical support for the idea that high calcium intakes may protect against high sodium intakes is provided by the study of Ilich *et al.* [20]. In a 3-year prospective study, postmenopausal women were randomly assigned to maintain usual sodium intake of about 3000 mg/day or to reduce intake to 1500 mg/day. All women also received calcium supplements, and total calcium intake averaged over 1300 mg/day. Because compliance with the sodium intervention was not high, results were reported by tertile of observed urinary sodium excretion rather than by initial group assignment. No negative associations between urinary sodium and bone density were observed [20]. This suggests that, at least in postmenopausal women with high calcium intakes, sodium intake does not adversely affect bone. 

Most women, however, have calcium intakes well below 1300 mg/day, and the estimated prevalence of inadequacy (intakes below requirements) is high. In both the United States and Canada, about 50% of premenopausal women had calcium intakes from food alone below the Estimated Average Requirement of 800 mg/day [21], as estimated in NHANES 2001-2002 and the 2004 Canadian Community Health Survey Cycle 2.2 [22,23]. To provide context for the potential impact of our findings for women with low calcium intakes, we used our data to predict the effect of reducing sodium intake from our participants’ average intake of 2942 mg/day (based on 24-h urinary excretion) to the Institute of Medicine’s Tolerable Upper Intake Level of 2300 mg/day [24]. Using the regression equation derived from our data (Urine Ca (mg) = 80.5 + 0.019 × urine sodium (mg)), urinary calcium excretion would be predicted to decrease by 12 mg/day. Assuming no compensatory changes in absorption, that would translate to preventing a loss of about 4 g of calcium on an annual basis (12 mg/day × 365 day/year), or 10 g of bone mineral content, given that bone mineral is about 40% calcium by weight. Whole body bone mineral content of our participants averaged 2300 g; thus, preventing a loss of 10 g equates to a 0.4% bone sparing effect over one year. Clearly, this estimate is fraught with assumptions, but if sustained over decades, suggests that the impact on fracture risk could be meaningful. 

Strong experimental or prospective evidence is not available regarding whether sodium intake impacts on bone health in younger women, and the available data are mixed. We found an association between urinary sodium excretion and bone density at the hip in women with calcium intakes below the median of 506 mg/1000 kcal. Supportive data were also obtained by Jones *et al.* [25]: In a combined group of pre-and post-menopausal women, they reported that urinary sodium correlated positively with urinary deoxypyridinoline, a bone resorption marker, and was negatively correlated with BMD in unadjusted but not adjusted analyses. Conversely, in a crossover trial of high *versus* low sodium intake for one week, no changes in bone resorption or calcium absorption were observed in 11 premenopausal women, although urinary calcium excretion increased [26]. Similarly, in a randomized seven-week trial of reduced sodium intake that included 15 young women (as well as 14 men), urinary sodium decreased but no group differences were seen in serum bone turnover markers [27]. The small sample sizes and short duration of these studies, however, may have limited their power. 

Although our sample size was reasonably large, our study also has limitations. Most notably, we had only a single measure of 24-h sodium excretion, and multiple collections are necessary to determine an individual’s “usual” excretion (and thus intake) with desirable accuracy. Although food frequency questionnaires estimate usual nutrient intakes, and sodium excretion was correlated with sodium intake as estimated from the food frequency questionnaire, the association was weak. The Diet History Questionnaire (the FFQ we used) has been validated [28,29], but dietary assessment of sodium intake is notoriously challenging. This is largely due to the varying levels of sodium in different varieties of similar products: for example, while different brands or varieties of a particular food (such as tomato sauce for spaghetti) have generally similar energy, macronutrient, and vitamin contents, the sodium content can vary dramatically, by well over 100% [30]. This is true for almost all foods that have been “processed” to any degree (e.g., foods other than raw fruits and vegetables, unprocessed grains). Furthermore, the FFQ does not assess addition of salt at the table, or in the preparation or cooking of foods. For these reasons, 24-h urine collections are still recommended for assessment of sodium intake, but multiple collections are desirable to capture day-to-day variability in intake.

While our results add to the suggestive evidence that the combination of high sodium intakes and low calcium intakes has adverse effects on bone, controlled trials are needed to provide more definitive conclusions.
